# Enhanced detection of *Opisthorchis viverrini* infection: A comparative evaluation of modified one-step FECT and conventional diagnostic methods in low-intensity setting

**DOI:** 10.1016/j.parepi.2024.e00391

**Published:** 2024-11-10

**Authors:** Suksanti Prakobwong, Lakhanawan Charoensuk, Kacha Chedtabud, Somchai Pinlaor, Srisupaph Poonlaphdecha, Alexis Ribas

**Affiliations:** a Department of Biology, Faculty of Science, Udon Thani Rajabhat University, Udon Thani 41000, Thailand; b The Parasitology, Geoinformatics, Environment and Health Science research group, Faculty of Science, Udon Thani Rajabhat University, Udon Thani 41000, Thailand; c Department of Clinical Pathology, Faculty of Medicine Vajira Hospital, Navamindradhiraj University, Bangkok 10300, Thailand; d Department of Geoinformatics for Development, Faculty of Humanities and Social Sciences, Udon Thani Rajabhat University, Udon Thani 41000, Thailand; e Department of Parasitology, Faculty of Medicine, Khon Kaen University, Khon Kaen 40002, Thailand; f Cholangiocarcinoma Research Institute, Khon Kaen University, Khon Kaen 40002, Thailand; g Parasitology Section, Department of Biology, Healthcare and Environment, Faculty of Pharmacy and Food Science, Institut de Recerca de la Biodiversitat (IRBio), University of Barcelona, Barcelona, Spain

**Keywords:** *Opisthorchis viverrini*, Light infection, One-step FECT, High sensitivity

## Abstract

The formalin-ethyl acetate concentration technique (FECT) is one of the most sensitive diagnostic method not only for all helminths, but also for *Opisthorchis viverrini* infections in stool examinations. However, it remains a diagnostic problem for light infections. We modified the one-step FECT to determine the low-intensity of *O. viverrini* infection and compared with various conventional detection methods. The study utilized 160 egg-positive and 160 randomly negative stool samples for *O. viverrini* eggs by conventional FECT (cFECT) to compare the methods, including the simple smear, the Kato-Katz method, the two commercial stool examination kits, and the one-step FECT. Our results showed that the one-step FECT method had the highest sensitivity (95.6 %), followed by cFECT (87.9 %), the Kato-Katz (55.5 %), Aquisfek SF-FIX® (48.3 %), simple smear (42.3 %), and Mini Parasep® SF (35.1 %). The ability of one-step FECT exhibited better ability to detect low parasite intensities compared to the cFECT (18 eggs per gram (e.p.g.) versus 34 e.p.g.) and the other conventional diagnostic methods. In addition, the investigation of *O. viverrini* infection in endemic regions in northeastern Thailand based on 3900 fecal samples revealed that the one-step FECT with an intensity of 66.8 e.p.g. (range 18–226) was significantly higher in sensitivity than cFECT, which had an intensity of 58.0 e.p.g. (range 34–214). Interestingly, fecal samples with less than 50 e.p.g. could not be detected by cFECT in 67 % of cases, and 69 out of 3900 samples were negative. In conclusion, one-step FECT improves the detection of low-intensity *O. viverrini* infection, which is suitable for parasites screening, especially for low-intensity infections in the community.

## Highlights

• Diagnostic methods for the detection of *Opistorchis viverrini* are challenged by decreasing parasite intensity in populations.
• Fecal samples were collected from endemic areas for *Opisthorchis viverrini* in Thailand.
• Six parasitological techniques, including the new one-step FECT, were compared for *O. viverrini* detection.
• The conventional FECT was compared with one-step FECT in 3900 individuals.
• In our field study, the one-step FECT demonstrated higher sensitivity and detected low-intensity infections than cFECT.

## 1 Introduction

Opisthorchiasis, caused by infection with the liver fluke *Opisthorchis viverrini*, is a health problem in the Greater Mekong subregion and has been particularly associated with the risk of the vital cancer, cholangiocarcinoma (CCA) (Sripa and Pairojkul, 2008). In Thailand, recent epidemiological data indicate that *O. viverrini* infection continues to persist (Sripa et al., 2021). Despite the national program to control *O. viverrini* infection during 2016–2021, various projects have been funded and implemented by government agencies to eradicate the parasitic diseases in targeted areas. Although the prevalence of *O. viverrini* infections decreased slightly, as shown by a previous survey (Sripa et al., 2021), new cases with low parasite intensity and reinfections still frequently ocurred (Prakobwong and Suwannatrai, 2020). In our previous survey in 2017 in the same target area as the present study, the intensity ranged from 20 to 10,000 e.p.g. and intensities above 100 e.p.g. were frequently found (Prakobwong et al., 2017a; Prakobwong et al., 2017b). However, the recent national survey estimated lower infection rates <5 % across Thailand, on the contrary, the incidence of CCA and the mortality rate of the disease were not declining (Sripa et al., 2021). The observed trend of decreasing parasite intensities renders the government-recommended Kato-Katz diagnostic technique inappropriate, as its sensitivity decreases at low levels of *O. viverrini* e.p.g. This decrease in parasite load necessitates adaptation of diagnostic methods to this new epidemiological situation.

Stool examination is still the standard method for *O. viverrini* infection in humans (Worasith et al., 2015). Common microscopic methods for diagnosing parasitic infection with *O. viverrini* in the gastrointestinal tract include the simple smear, formalin-ethyl acetate concentration technique (FECT), Kato-Katz, and commercial detection kits, with detection sensitivities ranging from 11.0 to 91 % (Charoensuk et al., 2019). The Modified FECT is commonly employed in hospitals and for intensive epidemiological studies, and it is considered the gold standard method (Worasith et al., 2015). However, the sensitivity for the detection of low-intensity parasite has not been sufficiently validated. Additionaly, a large number of fecal samples could not be carried and preserved under suitable conditions in the *O. viverrini* prevention and control projects.

Accordingly, we developed a one-step FECT to determine the low-intensity of *O. viverrini* infection and compared its efficacy with various conventional detection methods, including the simple smear, the Kato-Katz method, and the two commercial stool examination kits. In addition, the efficacy of one-step FECT for *O. viverrini* infection in endemic regions in northeastern Thailand was compared with the cFECT. The study may provide information on the selection of diagnostic methods for parasite screening and post-treatment, which could be useful for health education programs aimed to reduce the risk of hepatobiliary pathologies.

## 2 Materials and methods

### 2.1 Fecal samples collection and comparison of parasitological technique

Fecal samples were collected from May to June 2022 from villages in endemic areas around water resources in Mueang and Nong Han District in Udon Thani Province (northeast region of Thailand). Fecal samples were collected from 1800 individuals (746 males and 1054 females) aged 20 to 85 years, and all samples were diagnosed by cFECT. Subsequently, a sub-selection of samples was analyzed after obtaining cFECT results. The 160 egg-positive and 160 randomly selected negative samples for *O. viverrini* eggs were used to compare the techniques, including the simple smear, the Kato-Katz procedure, the two commercial stool examination kits, and the one-step FECT. For all coprological techniques, the final microscopic examination was performed at 400× magnification by two experienced investigators to detect and quantify the number of eggs.

#### 2.1.1 Simple smear technique

Approximately two grams of a fresh stool specimen were collected with a wooden stick and mixed with a drop of normal saline (0.9 %) on a glass slide with an applicator stick and stained by adding 50 % tincture of iodine. In cases where the stool was formed, material was taken from inside the stool specimen. The specimen was covered with a cover glass and observed under the microscope.

#### 2.1.2 Kato-Katz technique

One gram of feces was pressed through a mesh sieve to remove debris, and a portion was transferred to the template. After filling the hole, the template was removed, and the remaining sample was covered with a piece of cellophane previously soaked in a glycerol-malachite green solution, two slides were used. The number of eggs was counted under a light microscope and multiplied by 24 to calculate e.p.g. (Katz et al., 1972).

#### 2.1.3 Commercial stool examination kits

Two commercial kits were used, the Aquisfek SF-FIX® (Aquisel SLU, Spain) and the Mini Parasep® SF (AlcorFix, UK). The fecal examination protocol was performed according to the companies' recommendations and then observed under the microscope using one gram of feces.

#### 2.1.4 Conventional FECT

A quantitative FECT was slightly modified as previously described *(*Charoensuk et al., 2019*).* One gram of fresh feces was dissolved in normal saline and filtered through two layers of gauze. The volume of the sample was adjusted to 10 ml and centrifuged at 2500 rpm for 5 min. The pellet was resuspended in 7 ml of 10 % formalin and 3 ml of ethyl acetate and centrifuged at 2500 rpm for 5 min. The lipid floating in the supernatant was removed*.* After decantation, 2 drops of 10 % formalin was added to the sediment for microscopic observation*.*

#### 2.1.5 One-step FECT

The quantitative one-step FECT *(*see above*)* was modified as follows. One gram of feces was transferred to a 15 ml tube containing 11 ml of 10 % formalin to fix and store for 2 days. Before microscopic examination, 3 ml of ethyl acetate was added and mixed gently by hand, then immediately centrifuged at 2500 rpm for 3 min*.* After decanting the supernatant, 2 drops of 10 % formalin was added to the remaining sediment, all sediment was placed on the slide, and a coverslip was added for microscopic observation*.*

### 2.2 Comparative effectiveness of cFECT and one-step FECT in a field study

For this part of the study, we used a total of 3900 fecal samples collected from residents in the endemic areas of Northeast Thailand (this sampling is independent of the 1800 fecal samples used to compare all techniques). The effectiveness of detection was assessed by comparing the sensitivity of parasite detection methods at various egg quantity levels using both methods (cFECT and the one-step FECT).

### 2.3 Ethical approval and procedures for *O. viverrini*-infected participants

The research method for this study was approved by the Human Ethics Committee of Udon Thani Rajabhat University *(*HECUD.68*/*2022*).* Participants were informed of the research procedure and asked for their consent. We visited the villages involved in the study to explain the study. A qualified physician treated any parasite infections of the participants with effective medications, and all participants received health education about *O. viverrini* infection.

### 2.4 Data analysis

A positive individual for *O. viverrini* refers to the detection of at least one egg in the stool specimen examined by one of the six parasitological methods as previously described *(*Charoensuk et al., 2019*)*. The sensitivity and negative predictive value *(*NPV*)* of each parasitological examination technique were determined; for each coprological technique, data are presented as the average of the percentage and intensity of infection *(*e.p.g.*).* For the comparison in the field study, Student's *t*-test was used for parametric comparison of infection intensity between cFECT and one-step FECT. Statistical analyses were performed using SPSS version 23, and *p-*values of less than 0.05 were considered statistically significant. Agreement between one-step FECT and other parasitological methods and between intensity groups were calculated.

## 3 Results

### 3.1 Comparison of fecal examination methods for detection of *O. viverrini* eggs

**Fig. 1:**
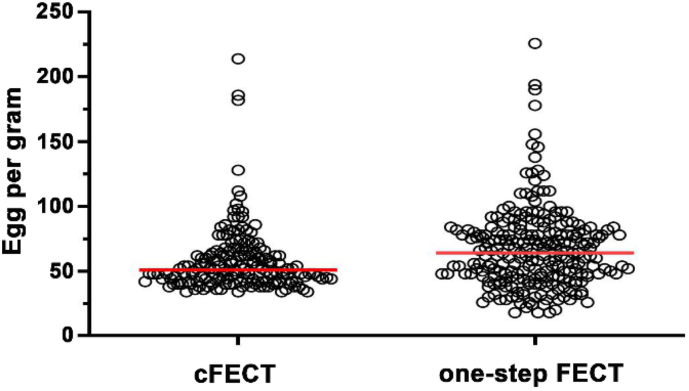
Comparison of e.p.g. distribution between the diagnostic techniques of cFECT and one-step FECT.

A total of 182 (160 positive cases were detected using the one-step FECT, and an additional 22 cases were detected using another techniques) of the 320 samples, including 160 samples that were initially positive and 160 negative samples after cFECT, were identified as containing *O. viverrini* eggs by at least one test method. We examined each fecal sample using six different diagnostic techniques, and our modified one-step FECT method consistently yielded the highest number of positive results, the highest sensitivity, and the highest NPV (Table 1). Specifically, cFECT identified *O. viverrini* eggs in only 160 samples, while the one-step FECT method detected 174 samples, resulting in NPV values of 86.2 and 94.5, respectively and a *p* value = 0.008. In addition, the one-step FECT method detected low intensity infections with a minimum of 18 e.p.g. In the same fecal samples, *O. viverrini* egg counts determined by the one-step FECT method tended to have a broader spectrum and higher mean value compared to egg counts determined by the cFECT method (Fig. 1).

**Table 1:** Comparison of the six stool examination methods for the detection of *O. viverrini* eggs.

Techniques	No. of positive	% infection (*n* = 1800)	Capacity of detection (e.p.g.) by One	Sensitivity	NPV	IRR Kappa value
	(*n* = 182)					
Simple smear	77	4.3	ND	42.3	56.5	0.582
cFECT	160	8.9	>34	87.9	86.2	0.932
One-step FECT	174	9.7	>18	95.6	94.5	0.976
Kato-Katz	101	5.6	>48	55.5	62.7	0.698
Aquisfek SF-FIX®	88	4.9	>42	48.3	59.4	0.64
Mini Parasep® SF	64	3.6	>50	35.1	53.7	0.501

ND: not determined.

### 3.2 One-step FECT improved the *O. viverrini* eggs detection in the field study

The cFECT and the one-step FECT method were compared for *O. viverrini* infection in 3900 individuals in endemic areas in the upper part of Northeast Thailand. The adapted one-step FECT yielded an average intensity of 66.8 eggs per gram (ranging from 18 to 226) across a total of 239 positive cases (6.1 %). In contrast, cFECT exhibited an average intensity of 58.0 e.p.g. (ranging from 34 to 214) with a total of 170 cases positive (4.3 %). The diagnosis performance of one-step FECT was significantly higher sensitivity than cFECT (*p* < 0.05). Interestingly, 67 % of subjects with low-intensity of *O. viverrini* egg less than 50 e.p.g. could not be detected by cFECT, and 69 out of 3900 samples were reported as false negatives (Table 2)*.*

**Table 2:** Comparison of sensitivity and *O. viverrini* egg counts using cFECT and one-step FECT in the field study. The intensity ranges are based on Charoensuk et al. (2019).

	one-step FECT				cFECT				Number undetected by cFECT (%)	IRR Kappa value
Range of intensity e.p.g.	No. of positive	% detection to 239 positive case	Median e.p.g. (range)	Sensitivity	No. of positive in same samples	% detection to 239 positive case	Median e.p.g. (range)	Sensitivity		
<50	82	34.30 %	40 (18–50)	91.8	27	11.30 %	36 (34–48)	58.8	55 (67.0 %)	0.484
51–75	74	30.90 %	64 (52–75)	93	62	25.90 %	48 (38–66)	81.6	12 (16.2 %)	0.908
76–100	63	26.30 %	84 (76–100)	98.5	61	25.50 %	58 (40–92)	95.5	2 (3.2 %)	0.983
>100	20	8.30 %	126 (104–226)	100	20	8.30 %	88 (62–214)	100	0 (0 %)	1
Total	239	100 %	66.8 (18–226)*	95.8	170	71.10 %	58.0 (34–214)	84	69 (28.9 %)	0.81

⁎ significant p < 0.05.

## 4 Discussion

The developed one-step FECT improves the detection of *O. viverrini* eggs in terms of e.p.g., sensitivity, and NPV compared to cFECT, especially in epidemiological contexts with low egg intensity in feces. Our results demonstrate that one-step FECT is consistently superior to other diagnostic techniques, including cFECT, simple smear, Kato-Katz, and commercial stool examination kits. In particular, the one-step FECT detected *O. viverrini* eggs in a significantly higher number of specimens and reduced false negatives. The higher sensitivity of the one-step FECT is reflected in the higher percentage of positive results and improved NPV. The NPV values of 94.5 % for the one-step FECT and 86.2 % for the cFECT highlight the superior ability of the one-step FECT to correctly identify uninfected individuals and reduce the likelihood of false-negative results (Brooker et al., 2005). In addition, the most notable finding of this study is that the one-step FECT can detect low-intensity infections with a minimum e.p.g. of 18.

Despite the advantages of the one-step FECT, certain limitations related to specimen manipulation in specific field studies might have arisen due to the inability to perform procedures on the same day. This was because a large number of fecal samples could not be transported and preserved under suitable conditions. Fixation of stool in formalin may reduce the viscosity of substances such as lipids or mucus in the sample (Ben Musa and Ibrahim, 2007). In addition, the presence of debris in some samples could impede egg observation. While gauze filtration, as employed in cFECT, is not feasible, a notable advantage of the one-step FECT is its ability to prevent egg attachment to the fibers of the gauze. At least 67 % of samples could not be detected by cFECT with fewer than 50 e.p.g. These results have important implications for improving the diagnosis and epidemiological understanding of *O. viverrini* in endemic regions (Sayasone et al., 2015). The ability of FECT to identify these cases in a single step may assist in ensuring the effectiveness of public health interventions, even when the level of infection is low.

Although commercially available kits generally have lower sensitivity than cFECT, Aquisfek SF-FIX® demonstrates comparable sensitivity values to Kato-Katz. Therefore, it could be considered a viable alternative to Kato-Katz in high e.p.g. situations. In certain situations, the Aquisfek SF-FIX® offers the advantage of preventing direct contact between the manipulator and the sample and reagents. Previous studies that compared commercial kits and other parasitological techniques in *O. viverrini* were restricted to the use of Mini Parasep® SF with an appropriate sample size (Kaewpitoon et al., 2016; Laoprom et al., 2016). The diagnostic sensitivity for opisthorchiasis is similar for all three techniques, but as previous studies did not use our modified one-step FECT, a comparison is not possible. Future studies should incorporate different techniques with appropriate sample sizes to obtain more robust data for low-intensity parasites that can be extended to other helminthiases.

Our results showed that *O. viverrini* infection persisted in all surveyed endemic areas in Thailand, with an infection rate of 6.1 %, and the overall prevalence of cFECT was estimated at 4.3 %. This value was higher than the recent estimate of 4.98 % in the Northeast (Sripa et al., 2021). Moreover, when considering individuals undetected by using cFECT and other techniques, especially those not treated with anthelmintic drugs, there remains a risk that residents may misunderstand and believe they do not have a disease. Accordingly, it was necessary to maintain and promote a comprehensive regional health education program for rural populations to reduce the risk of hepatobiliary pathologies, including CCA (Prakobwong and Suwannatrai, 2020). As previous mentioned, protective factors for CCA include knowledge, health beliefs, prevention behaviours, and community engagement (Songserm et al., 2022a; Sripa et al., 2017). All these factors can be improved through appropriate stool examination methods and health interventions to eliminate liver flukes in Southeast Asia (Songserm et al., 2022b). The one-step FECT and its ability to detect low-intensity infections can lead to more accurate epidemiological assessments and thus more targeted control and prevention strategies.

Recently, alternative methods for detecting liver fluke infections have been developed, such as serological tests, molecular and antigen detection methods (Boondit et al., 2020; Kaewpitoon et al., 2016; Worasith et al., 2023) and ultrasonography (Sungkasubun et al., 2016). Nevertheless, extensive field studies conducted in endemic areas often refrain from employing these new diagnostic methods due to constraints such as budget, equipment, and practicality. Additionally, some methods, such as antigen detection in urine (Worasith et al., 2023), have been developed recently and require an adaptation period for researchers. A limitation of our modified one-step FECT was the presence of a large amount of sediment that could interfere with visualization under the microscope. This requires the involvement of a skilled parasitologist with expertise in diagnosis.

## 5 Conclusion

Our study highlights the significant advantages of the one-step FECT method for detecting *O. viverrini* infections, especially light infections that were previously difficult to detect. This increased sensitivity has the potential to revolutionize the accuracy of epidemiological assessments, initiate more effective public health interventions, and ultimately contribute to reducing the risk of hepatobiliary pathologies associated with *O. viverrini* infections. The one-step FECT is a valuable tool in the ongoing encounter against this significant public health problem in endemic regions.

## CRediT authorship contribution statement

**Suksanti Prakobwong:** Writing – review & editing, Writing – original draft, Visualization, Validation, Supervision, Software, Resources, Project administration, Methodology, Investigation, Funding acquisition, Formal analysis, Data curation, Conceptualization. **Lakhanawan Charoensuk:** Writing – original draft, Methodology, Investigation, Formal analysis, Data curation. **Kacha Chedtabud:** Writing – original draft, Methodology, Investigation, Formal analysis, Data curation. **Somchai Pinlaor:** Writing – original draft, Methodology, Investigation, Funding acquisition, Formal analysis, Data curation. **Srisupaph Poonlaphdecha:** Writing – original draft, Methodology, Investigation. **Alexis Ribas:** Writing – review & editing, Writing – original draft, Visualization, Validation, Supervision, Resources, Project administration, Methodology, Investigation, Funding acquisition, Conceptualization.

## Declaration of competing interest

The authors declare that they have no known competing financial interests or personal relationships that could have appeared to influence the work reported in this paper.
